# How to Utilize my App Reviews? A Novel Topics Extraction Machine Learning Schema for Strategic Business Purposes

**DOI:** 10.3390/e22111310

**Published:** 2020-11-17

**Authors:** Ioannis Triantafyllou, Ioannis C. Drivas, Georgios Giannakopoulos

**Affiliations:** Research Lab of Information Management, Department of Archival, Library Science and Information Studies, University of West Attica, Ag. Spyridonos, Egaleo, 12243 Athens, Greece; triantafi@uniwa.gr (I.T.); gian@uniwa.gr (G.G.)

**Keywords:** app reviews, topics extraction, reviews classification, feature extraction methods, machine learning methods, text classification, text analysis, app business strategy

## Abstract

Acquiring knowledge about users’ opinion and what they say regarding specific features within an app, constitutes a solid steppingstone for understanding their needs and concerns. App review utilization helps project management teams to identify threads and opportunities for app software maintenance, optimization and strategic marketing purposes. Nevertheless, app user review classification for identifying valuable gems of information for app software improvement, is a complex and multidimensional issue. It requires foresight and multiple combinations of sophisticated text pre-processing, feature extraction and machine learning methods to efficiently classify app reviews into specific topics. Against this backdrop, we propose a novel feature engineering classification schema that is capable to identify more efficiently and earlier terms-words within reviews that could be classified into specific topics. For this reason, we present a novel feature extraction method, the DEVMAX.DF combined with different machine learning algorithms to propose a solution in app review classification problems. One step further, a simulation of a real case scenario takes place to validate the effectiveness of the proposed classification schema into different apps. After multiple experiments, results indicate that the proposed schema outperforms other term extraction methods such as TF.IDF and χ^2^ to classify app reviews into topics. To this end, the paper contributes to the knowledge expansion of research and practitioners with the purpose to reinforce their decision-making process within the realm of app reviews utilization.

## 1. Introduction

Over the last 10 years, mobile apps have tended to be the extension of capabilities that people have for completing their daily tasks and activities. Within this context, as the global number of smartphones increases, the number of app downloads from app-related stores is expanded and increased as well. According to Statista, in 2016 there were over 140.68 billions of mobile apps downloads, while at the end of 2019 downloads reached over 204 billion [1]. That is, a 45% increase among the examined years. This kind of rapid growth in digital economy is related with the intention of many enterprises to offer their products and services to their customers through mobile apps [2].

Mobile apps are distributed through platforms such as Apple Store and Google Play, while users can leave text reviews regarding their satisfaction from the apps. On the other hand, enterprises pay high attention to app reviews as a valuable source of information and feedback that could be analyzed and hence, develop potential actionable knowledge for apps optimization. This information is valuable to all the different operational dimensions within an organization [3]. Negative ratings and reviews can be exploited by the development team of an organization to fix several features such as bugs, interface issues, privacy, and security concerns, or even requests and ideas for potential app functionalities. Positive ratings and reviews could be exploited by a marketing team to include the strong functional points of an app within the next marketing strategy and its implementation. In addition, neutral ratings and reviews could be exploited by the whole project management team, diving into deeper corpus analyses, highlighting and tracking app issues, or supporting decision making in requirements for the prioritization process [4].

Nevertheless, app user review classification for identifying valuable gems of information for app software optimization is a complex and multidimensional issue. It requires sophisticated combinations of text pre-processing, feature extraction and machine learning methods with the purpose to classify app reviews into specific topics. Beforehand, a manual-reading approach of each one review separately is not a feasible solution [4,5,6]. That is, the more popular an app becomes, the more the reviews and hence the higher the complexity to analyze the reviews. Several prior efforts indicate automated feature engineering schemas that are capable to classify reviews per ratings or per general topics [4,5,6,7,8]. These efforts yielded significant indications, but they do not propose a feature engineering schema that is able to reduce the volume of reviews that app developers have to deal with, reinforcing in this way faster and more well-informed decision making [8]. Against this backdrop, we propose a novel one combination of feature engineering methods that classify with efficiency the examined dataset of app reviews into 12 specific topics. For this reason, we propose a novel feature extraction method, namely the DEVMAX.DF. This method is capable to detect earlier in smaller vector size of words within a review, a sufficient correlation importance for the topic that the review belongs to.

At the initial stage, pre-processing steps are taking place through the examined app reviews dataset such as tokenization, punctuation and stopwords removal and words stemming. After pre-processing, we use three different feature extraction methods. TF.IDF, χ^2^ (Chi2) and the proposed novel one DEVMAX.DF are involved, while examining a comparative analysis among them. Sequentially, multiple machine learning classification methods were deployed such as Naïve Bayes Multinomial, Logistic, SMO, IBk (kNN), J48 and Random Forest to identify the best possible combination of a feature engineering schema to classify efficiently app reviews into specific topics. One step further, to ensure the validity of the proposed classification schema, we proceed into the development of a real-case scenario, while examining 10 apps in order to classify their reviews into specific topics based on the proposed novel one schema.

To this end, the paper is organized as follows: In the next section, the importance of app reviews utilization and the related research efforts are unfolded within the realm of app reviews classification. The contribution of the paper is also explained furtherly while defining some research gaps. In Section 3, we describe the materials and methods that we use in this study. This contains the description of the dataset and its visualization, the text handling and word stemming, the different feature extraction methods that had been used, and lastly the deployed machine learning classification methods. Section 4 includes the proposed methodology while developing a practical problem statement that can be solved based on our proposed feature engineering schema. The process of schema selection and the evaluation criteria are unfolded into this section. Lastly, in Section 4, we describe how the simulation of the real-case scenario in classifying reviews of 10 apps into topics will take place. In Section 5, the results of our experiments are taking place. Finally, the Section 6 discusses the results and the practical contribution of the paper, while setting future research directions.

## 2. Related Background

### 2.1. Importance of App Reviews

Acquiring knowledge about users’ opinion and what they say regarding specific features within an app constitutes an initial and solid steppingstone for understanding their needs and concerns [3,5]. From an operational point of view, the engineering team is capable of understanding and comparing the frequency of each request that appears within the corpus of reviews; for how long each request on specific features appears; and if the frequency of the requests increases or decreases. This gives a practical evaluation of app performance while supporting engineers to monitor users’ satisfaction and apps’ health conditions in specific periods of time [6].

Furthermore, marketing efforts regarding the app’s promotion are stimulated and reinforced. That is, the utilization of app reviews is able to eavesdrop users’ request and ideas and thereafter provide more personalized marketing strategies to them through apps’ content [7]. In addition, in terms of app monetization, text reviews hide meaningful information regarding possible ads irrelevancy that unfortunately increases the users’ complaints, causing uninstallations and churns, and thus, apps’ retention decrease.

Generally, app reviews analysis and classification into specific features signalizes a contribution to the well-informed decision-making processes within the business context. As such, the app maintenance, optimization, and evolution [8]; the development of a roadmap to prioritize users’ requests [9]; and of course, the increase of apps’ retention within the users’ smartphones. This is a vital point, as app downloads from app stores might have increased rapidly, however, app retention rate never increased more than 39% from 2012 until 2019 [10].

### 2.2. Prior Efforts and Research Gap

Prior research approaches indicate a significant contribution into the realm of app reviews utilization with the purpose to extract useful knowledge in requirements engineering and for apps optimization. The paper [11] proposed MARA (Mobile App Review Analyzer) that extracts users requests from reviews through linguistic rules using Latent Dirichlet Allocation in order to identify feature requests and support prioritization strategy of app improvement.

In a similar vein to the linguistic-based approach, Johann et al. proposed the SAFE (Simple Approach for Feature Extraction) schema for extracting both app features from reviews and app descriptions for potential comparison purposes [12]. Maalej and Nabil proposed a novel schema of app reviews analysis based on probabilistic techniques in terms of classification [13]. They classified through their examined dataset four main categories, namely the bug reports, the feature requests, the ratings, and the user experiences. They used a binary approach while adopting supervised ML methods such as Naïve Bayes, Decision Trees and MaxEnt.

Relying on keyword-based approaches, MARK (Mining and Analyzing Reviews by Keywords) [14] proposed a semi-automated context that extracts keywords and associates them with negative reviews through a vector-based semantic representation. In the same line, Vu and colleagues [15] extract phrases from app reviews that are related with a negative sentiment and, thereafter, proceed into the visualization of sentiments and how they change over time. In an integrated approach from initial pre-processing through the end-to-end evaluation of the proposed conceptual model and involvement of developers in testing, SURF (Summarizer of User Reviews Feedback) proposes sophisticated summarization techniques for the sake of requirements engineering process including several topics for improvement [16].

Guzman and colleagues proposed an ensemble framework of algorithms and techniques for app reviews classification into categories for potential software optimization [17]. The results showed that the ensembles outperformed individual classifiers with an average precision of 74% and recall up to 59%. Subsequently, the researchers of the work [8] used deduced taxonomies to classify app reviews that are relevant with app maintenance and evolution. Following a merged approach of NLP, text and sentiment analysis, they achieved significant results of 74% in terms of precision and 73% of recall respectively. Within the realm of tweet classification, the study [18] relied on tree-ensemble methods of Random Forest (RF) and Adaboost Classifier (AC) while achieving notable accuracy results of 78.9% and 79.1% with two different feature extraction techniques, namely the Voting Classifier and TF.IDF respectively. This result is furtherly reinforced by recent notable findings of the same team referring that tree-based ensemble models perform well on text data and hence to the process of categorization app reviews [19].

Although these are noteworthy research findings and practical suggestions for the related community, to the best of our knowledge there is no a prior feature engineering method that is capable of identifying more efficiently and earlier terms/words within reviews that could be classified into specific topics. More specifically, there is major need to propose a novel feature engineering schema that is able to identify into smaller vector-sizes of words a sufficient correlation importance for the topic that a potential app review might belong to. That will practically help app administrators not to drop-out reviews that are not composed of too many words, and thus, exclude them from their app reviews datasets and experiments.

In addition, it is noted that even though there are significant results in app review classification per topic, the majority of them proceed into a broader approach while classifying reviews into major categories [13,20,21,22]. Broader classification approaches entail risks in their utilization such as the increase of the volume of the reviews that the project management team has to deal with; even they have already been classified. This kind of situation would increase the complexity in app requirements prioritization, delaying the decision-making process and probably reducing its quality [8]. Against this backdrop, we deploy and validate the proposed feature engineering schema into multiple sub-classes/topics that app reviews might address to. This will help practically to handle in a more explicit way the emerging technical issues and hence monitor apps’ health condition and maintenance more efficiently [12].

In continuation of the aforementioned research gap, it is noted that prior efforts focus on a triangular evaluation approach to identify the performance of their proposed feature engineering methods through the metrics of Precision, Recall and F-score (F1) [13,17,18,19,20,21]. However, it needs be mentioned that a further sensitiveness could be assigned into the evaluation metric of Precision, ensuring in this way that the examined app reviews are assigned properly into specific topics. Controversially, if the appropriate attention is not paid to the Precision rates, then this fact will lead unfortunately to reviews classification into wrong topics. In this respect, apart from well-established F1, we deploy another complementary evaluation metric, which is the product of Precision and F1. The purpose behind that choice is to cover the emerging research need of setting extra attention to the importance of Precision within the realm of app reviews classification.

In the next chapters, we propose the material, methods and methodology to tackle the addressed issues.

## 3. Materials and Methods

### 3.1. Dataset Description and Pre-Processing

The dataset that was used in our research obtained from GitHub and it was firstly deployed in a prior research effort at the same topic of app reviews utilization [23,24]. The whole dataset consisted of 7753 total reviews with multiple different variables/fields (7754–1 record in the “A Comic Viewer” app, which was completely empty). These include the review’s id, the name of the app, the app version, the text of the review, review’s author, review’s date, ratings from 1 to 5, the pre-classes, and sub-classes that corresponded to the topic of each review. We excluded some variables as they could not give a practical value to the research purpose of the paper. These variables are the review’s id, the app version, the review’s author, and the review’s date. We finally selected and elaborated the variables of the name of the app, the text of the review, the rating of each review, and the sub-classes that each review belongs to. Hereinafter, the subclass’ labels of the dataset are referred to as topics.

**Figure 1:**
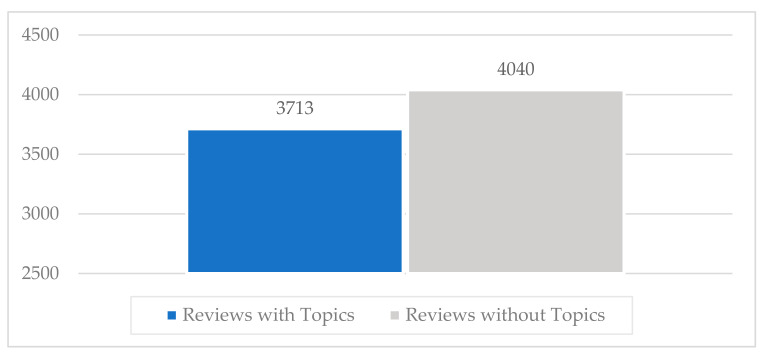
Number of reviews with topics and number of reviews without topics, respectively, derived from the total number of 7753 reviews. As can be seen, there are 4040 reviews without topics and 3713 with topics respectively.

Some reviews do not belong in a sub-class, that is, they do not have a topic. It is notable that the number of reviews without a topic were more than the number of reviews with topics (Figure 1). However, we decided to include both of them with the purpose to test and evaluate our proposed feature extraction schema under a noisy context of dataset. The involved sub-classes related with different and specific issues that users face when using the app. We selected the sub-classes and not the pre-classes of the whole dataset. The sub-classes could give more precise information to the owners of the app regarding its potential improvement. This also gives the opportunity to test the efficiency of the proposed feature extraction schema while having thousands of reviews that need to be classified into multiple specific topics.

It is noted that at this situation there is already given information regarding the name of the app, the review text of the app, the review rating, and the already classified review in a specific topic. Nevertheless, if a user submits a new review, then the topic of the review will be missing information. In the next table (Table 1), a sample of the dataset and the involved variables are presented. The grey color within the sub-class/topic column indicates the potential missing information that needs to be classified when new reviews will arrive. In the next sub-chapter, the visualization of the dataset takes place.

### 3.2. Dataset Visualization

**Figure 2:**
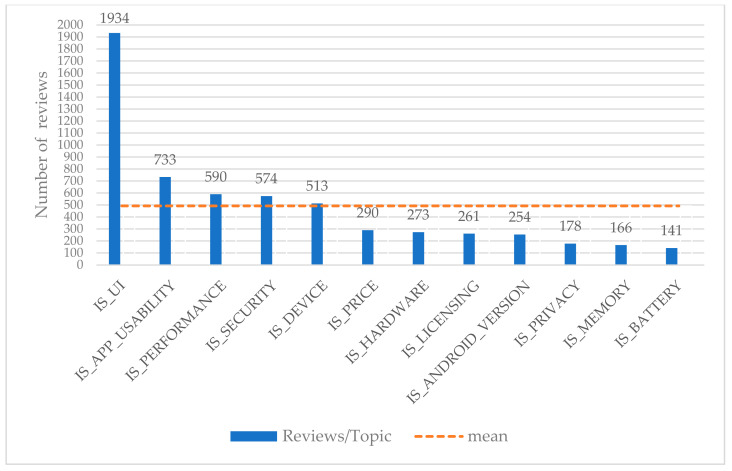
Number of reviews per topic. The whole examined dataset consists of 12 different topics and each one of them is related to a specific amount of reviews. Some topics include a higher number of reviews rather than others. User Interface (UI) topic yields the highest number of reviews (1934), App Usability articulates up to 733 reviews, Performance topic up to 590, and so on.

This sub-chapter represents the examined dataset graphically. To begin with, the whole dataset is composed of 7753 reviews. Some of these reviews have topics, while others do not. Figure 1 represents the number of reviews that have topics and those that do not have. Focusing on the reviews that have topics, one review might have one or more topic (one-to-many relationship). We depict the number of reviews per topic in Figure 2.

**Figure 3:**
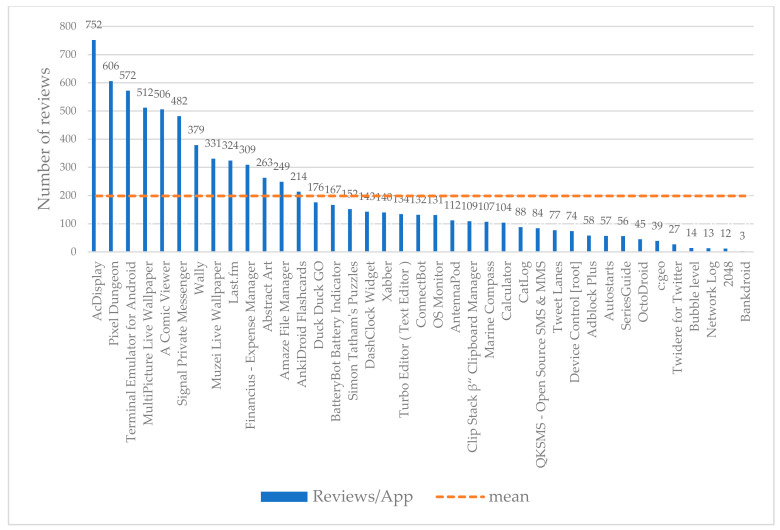
Number of Reviews per App. There are totally 39 different apps among the examined dataset. Each one of them has a different number of reviews.

**Figure 4:**
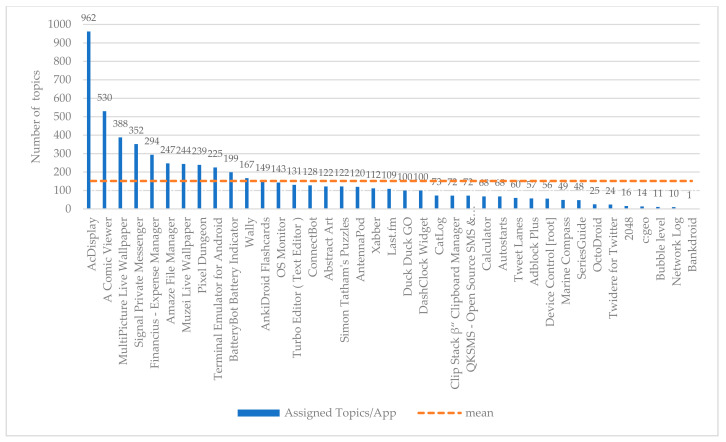
Number of assigned topics per app. As can be seen, some apps include more topics within their reviews rather than others.

Moreover, the reviews correspond to specific applications. In this respect, we depict in Figure 3 the allocation of reviews per app. Lastly, in Figure 4, the number of assigned topics per app are taking place. This is derived as follows: we take into consideration for each app the number of reviews that are related to this one app, and subsequently, for these reviews we have counted all the topics that are related to them.

### 3.3. Text Handling and Word Stemming

Preventing garbage-in garbage-out phenomena and trying to minimize the noise, we implemented further pre-processing for cleaning the app reviews. We adopt the techniques of tokenization, punctuation removal, upper-case conversion, and stop words removal. In addition, we applied stemming for reducing the words to their stem by eliminating the affixes [25,26,27,28]. The initial pre-processing was performed based on a text-handler [26] that is capable to perform transformations of app reviews texts into a suitable schema for the application itself. This achieves:

(A) the identification of textual units using trivial delimiters such as stops, spaces, question marks, etc. and
(B) the identification of extra-linguist elements such as abbreviations, list enumerators, numbers, dates, and so on.

After initial words pre-processing and identification, the stemming was performed. In this phase, the text-handler encapsulates the different morphological variations of words included within the app reviews. Subsequently, the word spotting process was implemented into two stages. First, the process of reducing the search space took place, and hence, optimized the performance of the text-handler. Based on statistical information, a small-size set of similar words has been extracted and grouped together under a common representative word. Secondly, an even more detailed procedure is implemented as the text-handler ranks the located words and outputs a complete “short-list” for each of the candidate words of the input text. Following prior research contribution [28], the score mechanism is based on a similarity estimator as can be seen below (Equation (1)):

*Similarity* (*W*1,*W*2) = *Common Position Trigrams* (*Left* (*W*1,*L*), *Left* (*W*2,*L*))/*L* (1)

*where L* = (*Length* (*W*1) + *Length*(*W*2))/2, *L* ∈ *N*

This estimator is designed to assign higher scores to morphological variations of the same root form using common position of trigrams. Based on prior research efforts, efficient grouping of words is articulated with a similarity score of 66.6% [28,29].

### 3.4. Feature Engineering Methods

Feature engineering constitutes one of the most important steps within the data preparation process. It leads to more efficient training of machine learning algorithms and therefore to an improved predictive performance [30,31]. In simplicity, feature engineering is the process of extracting new features that are derived from a raw dataset. In this paper, we use three different feature extraction techniques, the TF.IDF, the χ^2^ (Chi2 or Chi-square) and the proposed one of DEVMAX.DF.

a. **TF.IDF**

Term Frequency-Inverse Document Frequency (TF.IDF) constitutes one of the most classical feature extraction techniques in text analysis [32,33]. In simplicity, the TF.IDF assigns a weight to each term within a document based on its term frequency and inverse document frequency. In this way, the terms with higher frequency tend to be considered more important rather than others [31,32]. TF.IDF estimates the weight of each term by the above one equation (Equation (2)):

(2) $$\mathrm{TF}.\mathrm{IDF}=\mathrm{tf}*\log\frac{D}{DF}$$

*tf* is the frequency of term/feature f within the dataset, *D* is the total number of reviews and *DF* is the number of reviews containing the term/feature *f*.

b. **χ^2^**

The χ^2^ is another of the most-used feature extraction methods for text data analysis. It computes the probability of independence between the terms within a category [31]. Prior efforts indicate significant results regarding χ^2^ efficacy in text classification [34]. In a similar vein to [19], we test this feature extraction method to understand whether the occurrence of a specific term and the occurrence of a specific class are independent while analyzing the corpus. Therefore, we examine the dataset corpus of app reviews for each term, ranking them by their score following the equation (Equation (3)):

(3) $$\chi^{2}=\sum_{i=1}^{c}\frac{{\left(Oi-Ei\right)}^{2}}{Ei}$$

$$where\;Oi=DF_{i\;}\;and\;Ei=\frac{DF}{c}$$

Definitions of *DF_i_*, *DF* and *c* are presented in the following section.

c. **DEVMAX.DF**

In this section, we introduce a novel one text classification method, the DEVMAX.DF (Maximum Deviation). The core value of this method is to foster term-words that appear in one or more classes but not entirely [28,29]. The main purpose of the method is to promote the term/words in app reviews that have the maximum deviation in appearances, or alternatively, the minimum appearances in other classes from the basic class (max), that is, the class in which they mostly appear. In other words, DEVMAX.DF tries to promote term-words that are related mainly in one or more class (topic) and hence, articulate a sufficient correlation importance for the topic that the app review addresses. In order to promote the high appearance of term/words, the formula is articulated with a logarithm of the DF which is the number of reviews that include the term/word F (borrowed from TF.IDF). The DEVMAX.DF is described in the next equation (Equation (4)).

(4) $$\mathrm{DEVMAX}.\mathrm{DF}=\frac{\sqrt{\frac{1}{c-1}\;\sum_{i=1}^{c}{\left(\frac{DF_{i}}{D_{i}}-max\right)}^{2}}}{max}*log\left(DF\right)$$

$$where\;max=maximum_{i=1}^{c}\;\frac{DF_{i}}{D_{i}}$$

*DF_i_* is the number of app reviews that contain the term/feature *F* in class/topic *i*. Subsequently, *D_i_* constitutes the number of app reviews in class/topic *i*, and *c* is the number of classes/topics.

We provide a comparison among the selected feature extraction methods in the following chapters (V. Results), in a detailed manner.

### 3.5. Machine Learning Methods for Classification

Machine learning (ML) algorithms are commonly used within the context of classification problems [32]. In this study, we proceed into the adoption of six different binary ML algorithms to classify the app reviews into specific topics. The WEKA software was deployed for text classification. Written in Java, WEKA is a data mining and knowledge discovery software that is utilized both for practical business purposes and for research-related issues [3,35,36,37,38]. Among the selected ML methods, we used one Bayesian classifier, the Naïve Multinomial, two Function classifiers, the Logistic and the Sequential Minimal Optimization (SMO/SVM), one Lazy classifier, the IBk (kNN), and two classifiers from Decision Trees, namely the J48 and the Random Forest.

## 4. Methodology

At the initial stage of this section, we propose a practical problem statement. Thereafter, we unfold a proposed methodological approach to deal with the problem statement itself.

### 4.1. Practical Problem Statement

Company A owns an app in Google Play Store and help its users to cover their needs or to solve daily tasks and activities through this app. Google Play Store allows users to submit ratings and reviews for the app. These ratings and reviews are valuable sources of feedback for the company to understand issues that make users unsatisfied in terms of app usage and hence, setting priorities for improvement and optimization in specific features. In addition, the company wants to know and utilize the good ratings and reviews with the purpose to integrate them into the strategic marketing plan of the app and generally for promotional concerns. To this end, the company performs a text analysis into these reviews to find hidden valuable gems of information for improving the overall business strategy of the app and thus increase profit and recognition of its usefulness. To start with, the initial aim is to develop a methodological schema that is capable to classify the reviews into specific topics. That is, information about reviews and their ratings is available, however, the classification of app reviews into topics indicates the missing information. To solve this practical problem of app reviews utilization for business purposes, we combine the feature engineering methods that were proposed in Section 3.

### 4.2. Proposed Methodology

**Figure 5:**
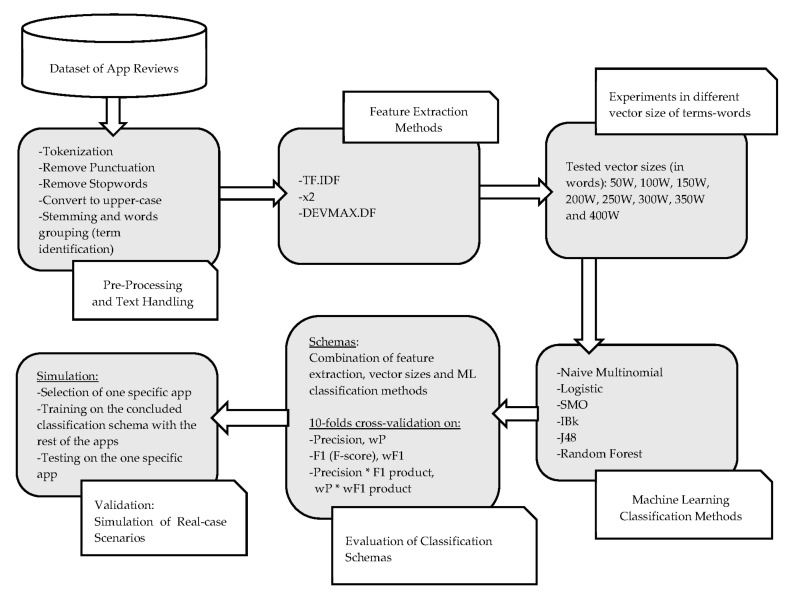
Methodology Flow Diagram.

In this paper, we use the combination of the aforementioned techniques that are described in Section 3. The purpose is to utilize the app reviews under a business-oriented context that was described in the practical problem statement. Figure 5 unfolds the proposed methodology.

### 4.3. Schema Selection Process

At the initial stage, we try to find through multiple experiments the most efficient feature engineering method to classify the app reviews of the examined dataset. In this phase, we do not take into consideration the apps particularly. This happens as the basic scope is to find the most accurate feature engineering schema to classify app reviews into specific topics.

First, pre-processing and text handling took place. Subsequently, we performed multiple experiments in different vector-sizes of term-words samples that consisted of 50, 100, 150, 200, 250, 300, 350, and 400 term-words in each feature extraction method, namely the TF.IDF, the χ^2^ and the DEVMAX.DF. The main purpose here is to understand which is the most efficient feature extraction method in text representation based on a specific range of vector-sizes of term-words.

It is also crucial to make comprehensible which ML classification method has the highest performance rating in classifying app reviews into topics. The 10 fold cross-validation method was used in all the ML classification schemas [20,29,39]. Binary representation of text vector space of the app reviews was used as a more efficient way rather than term-frequency representation for better performance of the examined ML classifiers [3,29,40].

### 4.4. Evaluation Criteria

In order to make comprehensible which feature schema has the highest performance, we used two evaluation parameters. The F1 (F-score) and the product of the Precision and the F1 (Precision*F1). The product of Precision and F1 was used as we are extra sensitive in the exactness of the proposed classification schema. For instance, if errors are detected in terms of app reviews classification into wrong topics, then wrong decision making could be taken by a potential project management team. Therefore, the product that derives from Precision and F1 is deployed for extra evaluation of the proposed classification schema. In addition, it is noted that we adopt the weighted mean of Precision (wP), F1 (wF1) and P*F1 product (wP*wF1). This derives and is calculated via the mean of Precision and F1 within the 12 topics, and it is weighted based on the population (number of appearances) of each topic in review. That is, the more the population of each topic, the more the weight and vice-versa.

### 4.5. Validation of the Selected Classification Schema

After the identification of the most efficient classification schema to classify app reviews into particular topics, we proceed into the validation process of the selected classifier at a specific app and its reviews among the rest dataset. The rest of the apps and their reviews were used for training purposes. At this point, someone could assume that the best app for classification of topics among the others is the one which has the highest number of reviews. However, as the basic scope of the proposed classification schema is to predict with efficiency the classification of a review into specific topics, then the emphasis was given for making experiments into apps that have the highest number of topics. That is, the highest population of topics to assign weights and not the highest population of reviews. As can be seen, this assumption is reinforced furtherly while taking into consideration the previous Figure 2 and Figure 4, namely the number of reviews per app and the number of assigned topics per app.

It is also worth noting that this approach constitutes a practical simulation of a real case scenario. That is, first we develop an efficient classification schema based on the analysis of an already existing corpus of app reviews. Therefore, if there is a “new” app with a new batch of reviews, then we deploy the classification schema into the new reviews as a test set and the rest of the already-existing apps reviews for training purposes. In this case, there is no practical value to predefine the percentages of training and testing such as 70% and 30% or 80% and 20%. In the next chapter, the results of our proposed methodology are unfolded.

## 5. Research Results

In this chapter, we present and discuss the results of our experiments. Different feature extraction and ML methods were used to make comprehensible, which is the most efficient classification schema for classifying app reviews into specific topics. First, performance results of selected feature extraction methods take place. Thereafter, we highlight the results of the most efficient classification schema in classifying app reviews into topics. Subsequently, results are presented regarding the efficiency of the selected classification schema into specific apps that are included within the dataset as practical use cases.

**Figure 6:**
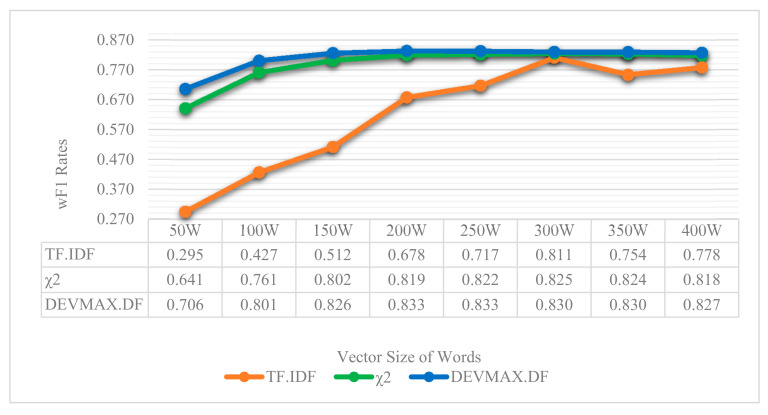
wF1 rates among the different feature extraction methods based on the most efficient classifier, the SMO, through different vector sizes of words.

**Figure 7:**
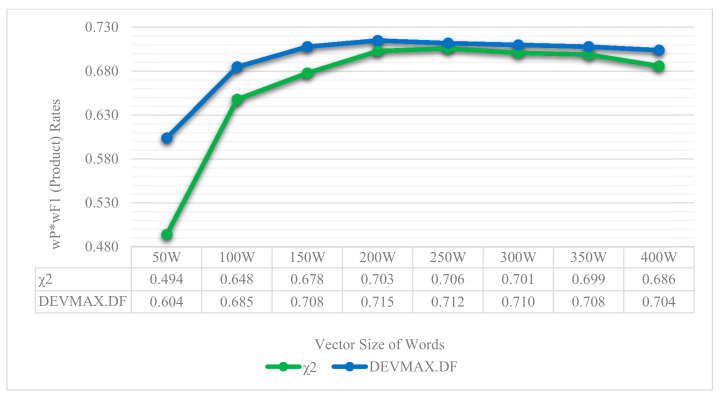
wP*wF1 product rates between the χ^2^ and the DEVMAX.DF feature extraction methods based on the most efficient classifier, the SMO, through different vector sizes of words.

In the next three tables (Table 2, Table 3 and Table 4), we present the results of each feature extraction method that was deployed. The vector size of the words is depicted horizontally, while the efficiency of each ML classifier is presented vertically. For space-saving reasons, we present the vector-sizes per 100 words, that is 100, 200, 300, and 400. However, for greater exactness, in the next figures (Figure 6 and Figure 7), the vector-size of words is presented per 50 words. The results are depicted through wP (weighted Precision) and wF1 (weighted F1) based on the appearances of topics in the reviews. That is, the weight is calculated based on the population of topics, which means how many times the topic appeared in reviews. More details and related results will be seen in Table 5.

The results of the experimental comparisons among the involved feature extraction methods and the deployed ML classifiers indicated that the best average values of wF1 and wP*wF1 product are met in DEVMAX.DF with SMO as a ML classifier. This schema extracts the highest performance gain of 85.8% in wP, 83.3% in wF1 and 71.5% at wP*wF1 product at the specific vector size of 200 words. Subsequently, Decision Tree J48 algorithm follows up in the same vector size of 200 words with DEVMAX.DF, reaching 87% in wP, 82.1% in wF1 and 71.4% in wP*wF1 product.

In terms of smaller vector sizes such as 100 words, results indicate once more that the feature extraction method of DEVMAX.DF performs better in most of the ML classifiers at classifying app reviews into specific topics. In addition, taking into consideration Table 2 and Table 3, TF.IDF and χ^2^ extracted lower performance rates especially in smaller vector sizes. This means practically that DEVMAX.DF, as a proposed feature extraction method, performs better in smaller-length reviews to identify words within them that represent a specific topic. We visualize these results in Figure 6 and Figure 7 where an even smaller vector size of 50 words depicts the greater efficiency of DEVMAX.DF compared to the TF.IDF and χ^2^.

At the same line, through most of the ML classifiers, DEVMAX.DF outperformed both TF.IDF and χ^2^ in bigger rangers of vector sizes such as 300 and 400 words. Controversially, there are very few occasions that χ^2^ barely outperforms DEVMAX.DF. Such as the vector size of 300 words, where χ^2^ with the Logistic classifier articulates a performance rate of wF1 with 75.9%, while DEVMAX.DF at the same instance resulted in a 75.2% wF1 rate.

Relying on the visualized results of Figure 6 and Figure 7, it is noted that in a smaller vector size of 50 words, the DEVMAX.DF manages efficiently to detect the best words for terms representation at an earlier phase rather than χ^2^ and TF.IDF respectively. For example, in Figure 7, χ^2^ yields a 49.4% wP*wF1 product value, while the DEVMAX.DF a 60.4% respectively. In other words, this highlights a significant improvement in smaller vector size of words of 22% while comparing χ^2^ and DEVMAX.DF as feature extraction methods. DEVMAX.DF achieves better results, 68.5%, also in 100 words with a percentage of improvement at 6%, while χ^2^ achieves 64.8%. These results are capable to cover recent research indications of applying the DEVMAX.DF into smaller texts [29].

**Figure 8:**
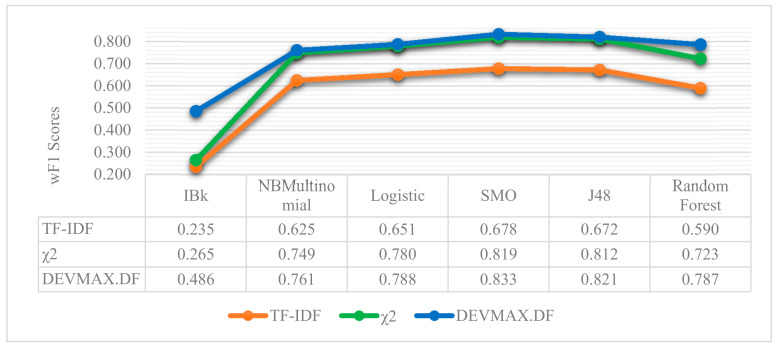
Machine learning classifiers and their performance in wF1 into the three different feature extraction methods based on the specific vector size of 200 W.

**Figure 9:**
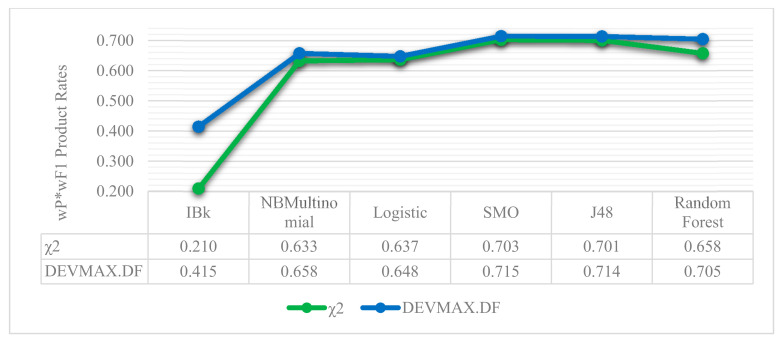
Machine learning classifiers and their wP*wF1 product performance rates between the χ^2^ and the DEVMAX.DF feature extraction methods based on the specific vector size of 200 W.

**Figure 10:**
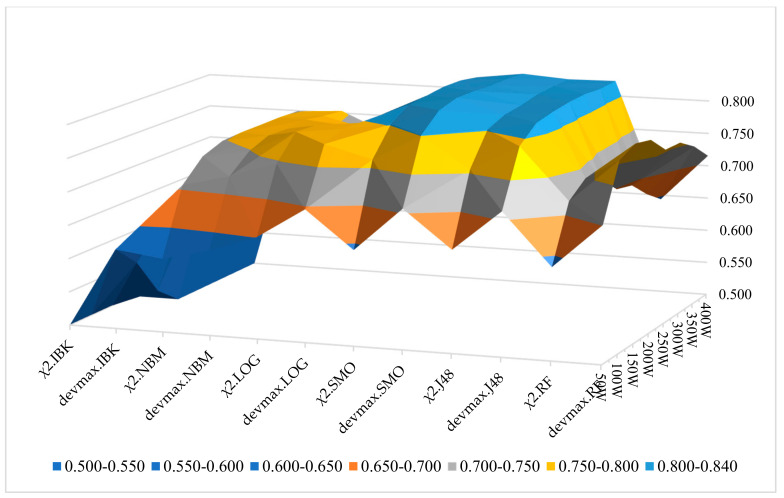
wF1 Solution Space for Classification Schemas.

Moreover, in Figure 8 and Figure 9, the performance of machine learning classifiers is presented in the three different feature extraction methods based on the 200-vector size of words. Additionally, in Figure 10, we present the wF1 solution space in terms of the local maxima that are yielded to the best performances, as represented in Table 2, Table 3 and Table 4. It is noted that in all the occasions of applying different ML classifiers, the DEVMAX.DF outperformed both TF.IDF and χ^2^.

As it was previously mentioned, based on the 200-vector size of words, the SMO algorithm in DEVMAX.DF has the greatest performance among the others with rates of wF1 and wP*wF1 product at 83.3% and 71.5% respectively. J48 follows up with 82.1% wF1 and 71.4% in wP*wF1 product. Random Forest constitutes the third best classifier with a performance rate of wF1 at 78.7% and wP*wF1 product at 70.5%. Naïve Bayes Multinomial, Logistic and IBk yielded lower performance rates, while the last one (IBk) indicated the lowest performance in classification schemas combinations.

Figure 10 is displaying the solution space of all the examined classification schemas and their wF1 rates on the 10-folds cross-validation technique. The best schemas (local maxima) are marked with light blue colour. In addition, this figure provides all the sets of the tested schemas’ combinations, and at the same time, the best performances of these tests. This also gives very useful information to upcoming researchers for re-examining their findings through these classification schemas, or preventing the examination in some of them if their corpus dataset is related with the purpose of this paper.

Therefore, based on the extracted results among the different combinations of feature extraction methods and the deployment of multiple ML classifiers, we select the most efficient classification schema, which is the DEVMAX.DF, in 200 vector size of words with SMO. In the next table (Table 5), the results of the most efficient classification schema in classifying reviews into topics are presented. In the left column, the 12 topics are depicted. Right next to this column, True Negatives (TN), False Negatives (FN), True Positives (TP), and False Positives (FP) are presented.

To evaluate the efficiency of the selected classification schema in classifying app reviews into specific topics, Precision and F1 are involved. The number of reviews per topic are presented in the last right column. It is noted that each topic articulates different sizes of reviews, and hence, different weightiness in topics classification efficiency. For this reason, the weighted means of Precision and F1 are presented in the last row of the table.

Till now, the greatest classification schema has been selected based on its performance in classifying app reviews in specific topics compared to the other schemas. Based on the proposed methodological approach, the next step is the practical testing and validation of the selected classification schema at specific apps. That is, we investigate the performance of the selected classification schema when classifying reviews in particular topics per app. In Table 6, Table 7 and Table 8, the results of three different apps are presented. The representation of these specific apps (“AcDisplay”, “A Comic Viewer” and “MultiPicture Live Wallpaper”) was selected based on their high population of topics compared to the rest of the apps, with lesser populations of topics within the examined dataset.

It needs to be mentioned that the current practical problem statement of classifying app reviews into certain topics, pays high attention to the Precision of each one classification result. As it was previously mentioned in Section 4, if there is no high attention in Precision as an evaluation criterion to correctly assign reviews into specific topics, then a project management team could lead to complexity of which reviews belong to specific topics. This will lead to setting wrong prioritizations in optimizing the app itself. Therefore, Precision is adopted once more as an evaluation metric in the next tables.

On the top left side of Table 6, Table 7 and Table 8, the name of the app is presented. At the same left column, the 12 topics take place. True Negatives (TN), False Negatives (FN), True Positives (TP), and False Positives (FP) are presented. Their sum is related with the total number of reviews in this specific app (see also in Figure 3). Weighted mean of Precision (wP) and weighted mean of F1 (wF1) rates are presented at the last row of the table. This happens as the populations of some topics in the reviews are bigger than others, and thus, their weightiness to the total weighted means.

Apart from the three apps that were presented with the purpose to prove the efficiency of the proposed classification schema into a real-case scenario, the last one table (Table 9) represents the top 10 apps with the highest population of topics among the examined dataset. In the last-right column of Table 9, the number of topics per app are depicted. At the last row of this column, the total number of topics per app for the first top 10 most populated apps is aggregated, that is 3680 (see also in Figure 4). It is notable that in most of the cases, there are very high rates of wP and wF1, indicating in this way that the proposed classification schema performs sufficiently in classifying app reviews into specific topics in a wider number of examined apps.

For instance, the Signal Private Messenger yielded a wP up to 85.88% and wF1 85.46% in classifying app reviews into topics. In almost the same line, Muzei Live Wallpaper articulates a wP up to 84.97% and a wF1 85.03% respectively. Lastly, the deployment of the selected classification schema into the app of the BatteryBot Baterry Indicator resulted in a wP of 92.60% and wF1 90.24%. These results are capable to verify the overall weighted mean of wP and wF1 among the examined apps, as it is depicted at the last row of Table 9.

**Figure 11:**
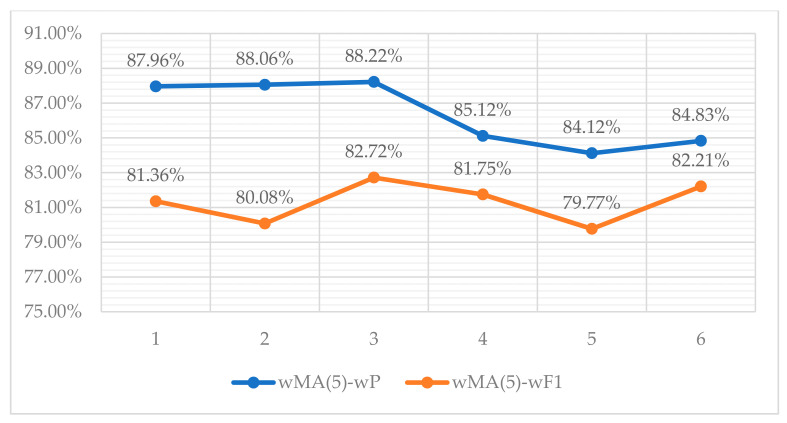
Weighted moving averages of wP and wF1 among five apps.

Lastly, in the next figure (Figure 11), we adopt the 5-point moving average, which is weighted by the number of assigned topics per app, hereinafter mentioned as wMA(5). This constitutes an indicator to visualize the performance of the selected classification schema in terms of the wP and the wF1 rates. The wMA(5) calculations are deployed to ensure statistical smoothness of the results among the different selected apps’ performance on topics classification.

Point 1 on Figure 11 indicates the weighted results of five apps starting from the first row of Table 9 (“AcDisplay” to “Financius-Expense Manager”); point 2 indicates the weighted results of five apps starting from the second row of Table 9 (“A Comic Viewer” to “Amaze File Manager”), etc. As can be seen in Figure 11, both two lines of wMA(5)-wP and wMA(5)-wF1 confirm the results of Table 9 in terms of the weighted means.

## 6. Discussions and Future Work

In this paper, we tried to classify app reviews into specific several topics based on a novel one feature extraction method, the DEVMAX.DF. Through multiple subsequent experiments and comparisons among the extracted results, we concluded that the best feature engineering and classification schema is articulated while using DEVMAX.DF with SMO machine learning algorithm in 200-vector size words. This combination resulted in the highest performance among the others, with up to 85.8% wP and 83.3% wF1, in terms of classifying app reviews into 12 different topics.

The proposed classification schema was tested under a noisy context of data as more than half of the whole dataset of app reviews were not labelled with topics (Figure 1). Against this backdrop, the proposed classification schema finally indicated a sufficient durability in terms of its discriminant capacity while performing efficiently in a fuzzy context of dataset. Therefore, we recommend this schema to be adopted in some already established theoretical attempts that ought to be evaluated for their practical accuracy within the realm of app reviews classification [3]. We also encourage other research approaches with significant results and indications to replicate their experiments while including the proposed novel one feature extraction method compared with others such as TF-IDF, χ^2^, Bag of Words, and so on [19,35].

Another significant and practical contribution of the proposed attempt is that the selected classification schema achieved better performance of reviews classification into smaller vector sizes of words. This means that if an app has multiple reviews but their length is limited with not enough vector sizes of words, then the proposed classification schema constitutes a reliable toolbox to classify app reviews into specific topics; even these reviews are lesser than 100 words, such as 50. More specifically, comparing the wP*wF1 product values of χ^2^ (49.4%) and DEVMAX.DF (60.4%) at the vector size of 50 words, an improvement of 22% with DEVMAX.DF is yielded in the process of classifying app reviews into topics (Figure 7). In other words, the proposed schema is capable to classify earlier in which topic a review should be assigned without waiting to have bigger-sized reviews such as 100, 150 or 200 words. This also prevents potential attempts that might exclude reviews from a dataset due to their low vector-size words. In this respect, to the best of our knowledge, there is no prior effort that proceeds the experiments and comparisons of different feature engineering schemas in the app reviews classification problem to earlier highlight the topic that a review belongs to; even if this review has smaller vector sizes of words. Of course, it is noted that in bigger vector sizes of words, the probability to have appropriate words in representation is increased, and therefore, the differences among the performance rates of the examined feature extraction methods are minimized. This assumption was proved and depicted in Figure 6, where both χ^2^ and DEVMAX.DF performed very closely to their rates in 250 w, 300 w, 350 w, and 400 w, while even the lowest-performed TF.IDF indicates increased rates in bigger vector sizes.

In addition, it was our choice to select and involve the sub-classes and not the pre-classes of the examined dataset. The purpose behind that choice is related to the effort to find a practical and specific solution for app developers to know more explicitly and not so broadly the potential technical issues of their apps, segmenting them into particular topics [13,20,21,22]. This will give a further advantage of knowing exactly what the problem is of an app, monitoring more constructively apps’ health condition [6], their maintenance [8], and setting more well-informed prioritization in terms of feature requirements [5,12].

Furthermore, the proposed attempt focused on the identification and development of a solution under a real-case scenario of classifying app reviews into topics for a set of multiple apps. The proposed classification schema was effectively able to classify reviews into specific topics for each of the examined apps individually (Table 6, Table 7, Table 8 and Table 9). For the sake of developing and implementing a real-case situation, we did not set a pre-defined approach of training and testing percentages such as 70–30% or 80–20% respectively. Controversially, we selected one app per experiment for testing the proposed classification schema, and the rest of the apps for training purposes. That is, under a practical business environment, if a team is assigned to solve an app review classification problem in an upcoming app of a new client, the reviews of the new app will be supplied to the concluded classification schema to categorize them into topics. The rest of the already existing app reviews datasets that the team has from prior app reviews classification problems will be set for training purposes. Based on the examined cases of this paper, the average training set was about 93% of the whole dataset, and the test set was about 7%.

It is also notable that in this paper we focused on the development and identification of an efficient classification schema, and we did not proceed into topics exploitation based on ratings. As it was mentioned within the practical problem statement, bad ratings of reviews could be assigned for optimizing the apps and good ratings could be utilized by a marketing team to enrich the strategic promotion of an app. Nevertheless, if there is no reliable, valid and consistent feature engineering schema at an initial stage that classifies the reviews into topics with accurate efficiency, then the rest of the sequential operational steps will be implemented in the wrong way. Wrong reviews will be assigned to wrong topics and complexity will be increased in terms of the frequency that they re-appear after rectifying users’ requests. For this reason, we demonstrated an extra sensitiveness at the Precision as an evaluation metric to ensure in this way that app reviews of the examined dataset are assigned properly into specific topics. More specifically, apart from the well-established F1 that is adopted in prior significant contributions in the app reviews classification realm [13,17,18,19,20,21], we involved a complementary evaluation metric, namely the Precision * F1 product. This will furtherly reinforce the effectiveness and the exactness of the proposed classification schema in classifying app reviews into topics, and hence, prevent the phenomena of assigning app reviews into wrong topics.

Regarding our future research efforts, until now, an efficient schema in app reviews classification problem has been developed and specified. We will start working sequentially on ratings classification. Till now, even our method indicated an analogous efficiency; we need to dive deeper into more methods and experiments. This will ensure the proper classification of topics based on their ratings for assigning them correctly within a project management team for app software optimization, feature requests of users, and/or marketing purposes. Furthermore, we will try to establish an even more integrated approach while including developers’ opinions [16] regarding the usefulness level of our proposed feature engineering schema. The involvement of app analytics metrics such as crashlytics occasions, retention percentages and downloads improvement, or time spent within the apps, would be practical supportive indicators to make comprehensible the efficiency level of the proposed classification schema [41]. Besides, this will help developers to foster a user-centred app life cycle, utilizing in this way the reviews by the users, for the users. That is, to reinforce the strategy of indirectly involving users in app optimization, and hence, positively impact the app success as a system [42].

Additionally, further research is needed to deploy the proposed schema into other realms of text representation and analytics. Sectors such as e-learning environments and learners reviews [43] or product reviews in online shopping industry [44] constitute another research domain that needs to be explored. Lastly, we need to state that the proposed novel feature extraction method of DEVMAX.DF is strengthened further based on the results of this paper, but also from prior research approaches [28,29]. However, even though there is a piece of research road that already has been travelled, more experiments through different datasets are needed in order to expand both the efficiency and reliability of our method.

## Figures and Tables

**Table 1:** Sample of dataset regarding a random app name, text reviews, ratings, and the topic that it belongs to. The last grey column denotes the potential missing information in case of a new review.

App_Name	Review Text	Review Rating	Sub-Class/Topic
ACDisplay	Good application it will be better if you make your app more smooth and add previews for each pics when locate the pages	4	User Interface
	It just shuts down without any warning! I use Android lollipop and everytime I open up anything it would just close down instantly. If it fix this problem I probably pay for the premium version.	1	Android Version AND Licensing
	Not for marshmallow Can’t access SD card for marshmallow.	2	Hardware

**Table 2:** Feature Extraction Results with TF.IDF.

Vector Size												
	*100 Words*			*200 Words*			*300 Words*			*400 Words*		
*ML Classifiers*	wP	wF1	wP*wF1	wP	wF1	wP*wF1	wP	wF1	wP*wF1	wP	wF1	wP*wF1
NBMultinomial	0.670	0.417	0.279	0.781	0.625	0.488	0.788	0.691	0.544	0.779	0.710	0.553
Logistic	0.628	0.440	0.276	0.749	0.651	0.488	0.733	0.694	0.508	0.698	0.693	0.484
SMO	0.536	0.427	0.229	**0.771**	**0.678**	**0.523**	0.811	0.746	0.605	0.832	0.778	0.647
IBk	0.466	0.218	0.102	0.628	0.235	0.148	0.701	0.230	0.161	0.740	0.227	0.168
J48	0.606	0.434	0.263	0.790	0.672	0.531	0.831	0.758	0.631	**0.857**	**0.798**	**0.684**
RandomForest	0.606	0.409	0.248	0.825	0.590	0.487	0.869	0.620	0.539	0.919	0.595	0.547

**Table 3:** Feature Extraction Results with χ^2^.

Vector Size												
	*100 Words*			*200 Words*			*300 Words*			*400 Words*		
*ML Classifiers*	wP	wF1	wP*wF1	wP	wF1	wP*wF1	wP	wF1	wP*wF1	wP	wF1	wP*wF1
NBMultinomial	0.873	0.706	0.617	0.845	0.749	0.633	0.825	0.750	0.619	0.806	0.749	0.604
Logistic	0.847	0.747	0.632	0.816	0.780	0.637	0.766	0.759	0.582	0.706	0.723	0.511
SMO	0.851	0.761	0.648	**0.858**	**0.819**	**0.703**	0.850	0.825	0.701	0.838	0.818	0.686
IBk	0.715	0.305	0.218	0.794	0.265	0.210	0.829	0.245	0.203	0.835	0.230	0.192
J48	0.868	0.755	0.655	0.863	0.812	0.701	**0.857**	**0.821**	**0.704**	**0.855**	**0.822**	**0.703**
RandomForest	0.865	0.718	0.621	0.910	0.723	0.658	0.921	0.690	0.636	0.934	0.644	0.601

**Table 4:** Feature Extraction Results with DEVMAX.DF.

	Vector Size											
	*100 Words*			*200 Words*			*300 Words*			*400 Words*		
*ML Classifiers*	wP	wF1	wP*wF1	wP	wF1	wP*wF1	wP	wF1	wP*wF1	wP	wF1	wP*wF1
NBMultinomial	0.890	0.723	0.644	0.865	0.761	0.658	0.841	0.762	0.641	0.818	0.753	0.616
Logistic	0.851	0.780	0.663	0.823	0.788	0.648	0.762	0.752	0.573	0.714	0.722	0.515
SMO	0.855	0.801	0.685	**0.858**	**0.833**	**0.715**	0.855	0.830	0.710	0.851	0.827	0.704
IBk	0.848	0.585	0.496	0.855	0.486	0.415	0.861	0.443	0.382	0.855	0.378	0.323
J48	0.873	0.793	0.692	0.870	0.821	0.714	0.862	0.825	0.711	**0.864**	**0.823**	**0.712**
RandomForest	0.863	0.781	0.674	0.896	0.787	0.705	0.901	0.759	0.684	0.910	0.715	0.651

**Table 5:** Results of the Most Efficient Classification Schema.

DEVMAX.DF-200W-SMO	TN	FN	TP	FP	Precision	F1	Number of Reviews per Topic
1-Privacy	7563	50	128	12	0.914	0.805	178
2-Hardware	7396	84	189	84	0.692	0.692	273
3-Device	7170	106	407	70	0.853	0.822	513
4-Performance	7099	56	534	64	0.893	0.899	590
5-Battery	7608	3	138	4	0.972	0.975	141
6-Price	7395	42	248	68	0.785	0.818	290
7-App_Usability	6911	168	565	109	0.838	0.803	733
8-Android_Version	7478	119	135	21	0.865	0.659	254
9-User_Inteface	5625	312	1622	194	0.893	0.865	1934
10-Licensing	7426	85	176	66	0.727	0.700	261
11-Memory	7565	11	155	22	0.876	0.904	166
12-Security	7093	62	512	86	0.856	0.874	574
**Weighted Mean:**					**wP 0.858**	**wF1 0.833**	Total: 5907

**Table 6:** Performance of the selected classification schema in classifying reviews into specific topics on the selected app, namely the **AcDisplay.**

App: AcDisplay	TN	FN	TP	FP	Precision	F1	Number of Reviews per Topic
1-Privacy	740	2	9	1	0.900	0.857	11
2-Hardware	685	42	15	10	0.600	0.366	57
3-Device	665	22	56	9	0.862	0.783	78
4-Performance	679	9	54	10	0.844	0.850	63
5-Battery	723	2	27	0	1.000	0.964	29
6-Price	733	1	8	10	0.444	0.593	9
7-App_Usability	686	14	35	17	0.673	0.693	49
8-Android_Version	718	14	20	0	1.000	0.741	34
9-User_Inteface	374	32	341	5	0.986	0.949	373
10-Licensing	744	1	1	6	0.143	0.222	2
11-Memory	744	0	5	3	0.625	0.769	5
12-Security	466	45	207	34	0.859	0.840	252
**Weighted Mean:**					**wP 88.56%**	**wF1 83.90%**	Total: 962

**Table 7:** Performance of the selected classification schema in classifying reviews into specific topics on the selected app, namely the **A Comic Viewer.**

App: A Comic Viewer	TN	FN	TP	FP	Precision	F1	Number of Reviews per Topic
1-Privacy	505	0	0	1	0.000	0.000	0
2-Hardware	458	18	21	9	0.700	0.609	39
3-Device	451	6	41	8	0.837	0.854	47
4-Performance	387	4	114	1	0.991	0.979	118
5-Battery	505	0	1	0	1.000	1.000	1
6-Price	491	7	7	1	0.875	0.636	14
7-App_Usability	436	14	51	5	0.911	0.843	65
8-Android_Version	491	8	7	0	1.000	0.636	15
9-User_Inteface	345	33	87	41	0.680	0.702	120
10-Licensing	491	5	7	3	0.700	0.636	12
11-Memory	407	64	34	1	0.971	0.511	98
12-Security	504	0	1	1	0.500	0.667	1
**Weighted Mean:**					**wP 86.17%**	**wF1 74.76%**	Total: 530

**Table 8:** Performance of the selected classification schema in classifying reviews into specific topics on the selected app, namely the **MultiPicture Live Wallpaper.**

App: MultiPicture Live Wallpaper	TN	FN	TP	FP	Precision	F1	Number of Reviews per Topic
1-Privacy	511	0	0	1	0.000	0.000	0
2-Hardware	501	4	6	1	0.857	0.706	10
3-Device	410	19	79	4	0.952	0.873	98
4-Performance	490	2	18	2	0.900	0.900	20
5-Battery	503	0	9	0	1.000	1.000	9
6-Price	504	0	6	2	0.750	0.857	6
7-App_Usability	472	17	19	4	0.826	0.644	36
8-Android_Version	484	11	17	0	1.000	0.756	28
9-User_Inteface	342	31	127	12	0.914	0.855	158
10-Licensing	507	2	3	0	1.000	0.750	5
11-Memory	505	0	6	1	0.857	0.923	6
12-Security	497	1	11	3	0.786	0.846	12
**Weighted Mean:**					**wP 91.50%**	**wF1 83.42%**	Total: 388

**Table 9:** Performance of the selected classification schema in classifying app reviews in specific topics for the top 10 examined apps with the highest population of topics in their reviews.

Apps	wP	wF1	Number of Topics per App
AcDisplay	88.56%	83.90%	962
A Comic Viewer	86.17%	74.76%	530
MultiPicture Live Wallpaper	91.50%	83.42%	388
Signal Private Messenger	85.88%	85.46%	352
Financius-Expense Manager	87.07%	77.30%	294
Amaze File Manager	90.96%	81.89%	247
Muzei Live Wallpaper	84.97%	85.03%	244
Pixel Dungeon	75.69%	78.25%	239
Terminal Emulator for Android	80.79%	76.60%	225
BatteryBot Battery Indicator	92.60%	90.24%	199
**Weighted Mean:**	**86.98%**	**81.62%**	Total: 3680
